# Knowledge and Attitudes of Community Pharmacists Towards Antibiotic Use and Antimicrobial Resistance in Western Greece

**DOI:** 10.3390/antibiotics15020184

**Published:** 2026-02-07

**Authors:** Maria Sarri, Despoina Gkentzi, Stelios F. Assimakopoulos, Markos Marangos, Maria Lagadinou

**Affiliations:** 1 Community Pharmacist, 26500 Patras, Greece; mar.sar2303@gmail.com; 2 Medical School of Patras, University of Patras, 26504 Rio, Greece; gkentzid@hotmail.com (D.G.); sassim@upatras.gr (S.F.A.); mmarangos@yahoo.com (M.M.)

**Keywords:** antibiotic use, antibiotics, pharmacists, resistance

## Abstract

Background: Antibiotic misuse and overuse remain a critical driver of antimicrobial resistance (AMR), a global health threat associated with increased morbidity, mortality, and healthcare costs. In Greece, where antibiotic consumption and resistance rates are among the highest in Europe, community pharmacists are well-positioned to contribute to antimicrobial stewardship efforts. Objective: This study aimed to assess the knowledge and attitudes of community pharmacists in Achaia, Western Greece, regarding antibiotic use and AMR, in order to identify knowledge gaps and inform future educational interventions. Methods: A cross-sectional survey was conducted between May and July 2023 among 207 pharmacists using a structured, self-administered questionnaire. The survey assessed demographics, knowledge of antibiotic indications, dispensing practices, and awareness of AMR. Statistical analysis included Chi-square tests and multivariate logistic regression. Results: Pharmacists demonstrated high levels of knowledge regarding appropriate antibiotic use in conditions such as sore throat (95%), bronchitis (76%), influenza (77.5%), and diarrhea (95%). However, knowledge was lower for rhinitis (60%) and sinusitis (56%). Almost all pharmacists (99%) were aware of AMR, and 86% perceived it as a significant public health issue in Greece. Logistic regression showed that pharmacists with 5–10 years of experience were significantly less likely to believe that antibiotics are always effective (OR = 0.08, *p* = 0.042). Conclusion: Pharmacists in Western Greece are generally well-informed about antibiotic use and AMR, yet misconceptions persist, especially for viral infections. Targeted educational interventions, interprofessional collaboration, and stricter enforcement of prescription regulations are needed to strengthen the role of pharmacists in combating AMR at the community level.

## 1. Introduction

Antimicrobials have changed global healthcare, saved millions of lives and become some of the most prescribed medications worldwide [1]. However, the growing misuse and overuse of antibiotics have significantly contributed to the emergence of antimicrobial resistance (AMR). The World Health Organization (WHO) estimates that 20–50% of antibiotics prescribed in primary care are used inappropriately [2], accelerating resistance development.

AMR is now considered one of the most pressing global health threats, resulting in approximately 700,000 deaths annually [3]. Infections caused by resistant pathogens lead to increased morbidity, mortality, and healthcare costs, highlighting the urgent need for effective measures [4,5].

In Greece, AMR is a particularly critical public health concern. According to the European Center for Disease Prevention and Control (ECDC), both antibiotic consumption and resistance levels in Greece are among the highest in Europe—in hospitals and the community alike [6,7]. Multidrug-resistant (MDR) pathogens such as *Klebsiella pneumoniae*, *Acinetobacter baumannii*, and *Pseudomonas aeruginosa* remain prevalent, with alarming resistance levels to last-line agents, including carbapenems [6,7]. Furthermore, infections due to methicillin-resistant *Staphylococcus aureus* (MRSA) and vancomycin-resistant *Enterococcus* (VRE) continue to strain the national healthcare system [8].

Outpatient antibiotic use in Greece remains elevated. Surveillance data from the ESAC-Net consistently rank Greece among the top countries in outpatient antibiotic consumption, measured in defined daily doses (DDD) per 1000 inhabitants per day [7]. Contributing factors include cultural norms, availability of antibiotics without prescription, and patient expectations for antimicrobial therapy—even in viral infections. Despite legal prohibitions, non-prescription antibiotic use persists, particularly in community pharmacy settings [9,10].

In response to this challenge, Greece has implemented several AMR surveillance and control strategies. Notably, it was one of the first countries to establish an electronic resistance monitoring network—WHONET-Greece—based on routine hospital susceptibility data [11]. More recently, Greece adopted a National Action Plan aligned with the WHO Global Action Plan on AMR, focusing on enhanced surveillance, antibiotic stewardship, and public education [12].

Pharmacists are increasingly recognized as key stakeholders in antimicrobial stewardship. Recent evidence from Eastern Mediterranean and neighboring settings (e.g., Egypt, Jordan, Palestine, Cyprus/North Cyprus, Saudi Arabia, and Lebanon) indicates that community pharmacists often face patient pressure and structural barriers that influence non-prescription antibiotic supply and stewardship practices [13]. Accordingly, Sullman et al. reported that pharmacists showed strict compliance with prescription-only regulations and described regulatory frameworks and electronic prescribing systems as a practical “protective mechanism” that supports refusal of inappropriate requests. They noted that these measures have shifted some misuse away from direct non-prescription demands toward attempts to reuse, prolong, or “top up” previous prescriptions or leftover antibiotics kept at home [14]. Pharmacists also reported frequently managing patient expectations for antibiotics in self-limiting conditions, using brief counseling approaches, written informational materials, and recommendations for symptomatic treatment to encourage appropriate antibiotic use [15]. Their accessibility and frequent interaction with the public place them in a unique position to promote rational antibiotic use, enhance treatment adherence, and educate patients on the dangers of misuse. While pharmacists generally acknowledge the importance of their role in combating AMR [15], knowledge gaps and inconsistent practices remain, especially considering that antimicrobial resistance is not included in undergraduate studies.

The aim of this study was not only to describe the knowledge and attitudes of community pharmacists in Achaia, Western Greece, regarding antibiotic use, overprescription, and antimicrobial resistance, but also to address three specific objectives: (a) to identify demographic and professional predictors of misconceptions regarding antibiotic effectiveness and AMR, (b) to generate evidence that may inform the development of targeted antimicrobial stewardship strategies and educational interventions in the community pharmacy setting, and (c) to position the findings within the broader European and Eastern Mediterranean context, where antibiotic consumption and resistance rates remain among the highest. The findings may inform the development of targeted educational interventions to support more rational antibiotic prescribing and dispensing practices in the country. Although several studies have assessed knowledge and attitudes toward antibiotic use and antimicrobial resistance among healthcare professionals in Greece and internationally, important gaps remain. Most Greek studies have focused either on hospital-based professionals or on the general public, with limited recent data specifically addressing community pharmacists, who represent a critical interface between the healthcare system and the population. Moreover, few studies have explored in depth how professional experience influences misconceptions regarding antibiotic effectiveness and antimicrobial resistance.

The present study adds to the existing literature by providing contemporary, region-specific data from Western Greece, an area with particularly high antimicrobial consumption and resistance rates. In addition to evaluating general awareness, this study uses detailed clinical scenarios to assess pharmacists’ knowledge regarding common community infections and examines demographic and professional predictors of misconceptions through multivariate analysis. Importantly, it investigates potential non-linear associations between years of professional experience and knowledge of antibiotic use and AMR, offering new insight into how knowledge may evolve across different career stages. These findings aim to support the design of targeted, experience-tailored educational interventions for community pharmacists.

## 2. Results

### 2.1. Demographic Characteristics

A total of 207 out of 350 pharmacists (59.1%) completed the questionnaire and were included in the analysis. The demographic characteristics of the respondents, including gender, age group, educational attainment, work sector, and years of professional experience are presented in Table 1. The sample included a nearly equal gender distribution (47.3% male, 52.7% female), and the majority (39.1%) were aged 41–50 years. All participants were employed in the private sector. Most held at least a university degree, with 36.7% holding a master’s degree and 6.3% a PhD.

### 2.2. Pharmacists’ General Knowledge and Attitudes Towards Antibiotic Use

**Figure 1:**
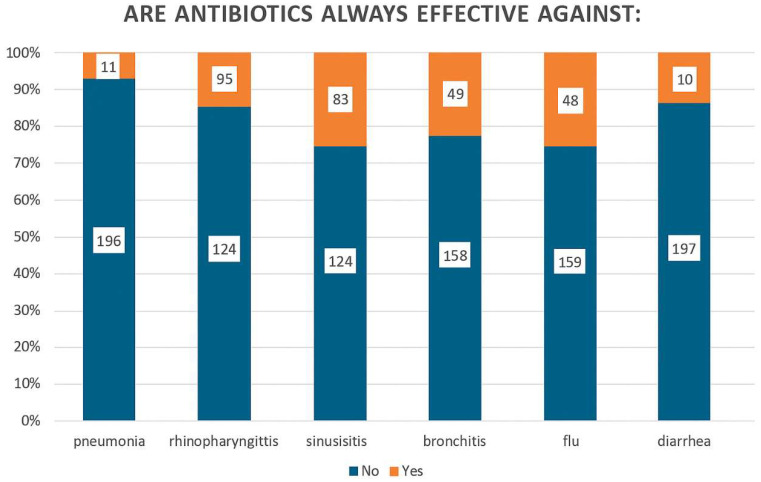
Pharmacists’ responses to the question “Are antibiotics always effective against the following conditions?”. Bars represent the proportion of respondents answering “No” (blue) and “Yes” (orange) for each condition (pneumonia, rhinopharyngitis, sinusitis, bronchitis, influenza, and diarrhea). Numbers within bars indicate absolute frequencies of responses. The figure illustrates that although most pharmacists correctly recognized that antibiotics are not always effective, notable misconceptions persist, particularly for upper respiratory tract infections such as sinusitis, bronchitis, and influenza.

General knowledge regarding antibiotic use was assessed through several questions, mostly clinical scenarios (e.g., factors leading to the unnecessary use of antibiotics, use of antibiotics for rhinitis or sore throat, etc.). The analysis of the responses indicates that pharmacists’ overall level of knowledge regarding the effectiveness of antibiotics was relatively high. Most participants recognized that antibiotics are not always indicated for the treatment of infections such as sore throat (95%), bronchitis (76%), influenza (77.5%), and diarrhea (95%). However, their knowledge was somewhat lower in cases such as rhinitis (60% correct vs. 38% incorrect) and sinusitis (56% correct vs. 44% incorrect) (Figure 1).

The possible associations between demographic factors (gender, age, educational level, and years of professional experience) and the level of knowledge were examined. The results are presented in Table 2.

A multivariate logistic regression analysis was performed to identify factors associated with the belief that “*antibiotics are always effective*” (regarding all infections asked). Male pharmacists provided more false responses than female pharmacists. However, this difference was only marginally significant (*p* = 0.059). Therefore, we cannot confidently conclude that there is a real gender difference in antibiotic use knowledge. Moreover, pharmacists with only a basic degree did not have a significantly different number of errors compared to those with a postgraduate degree (*p* = 0.847). Finally, after adjusting for age, gender and educational level, years of professional experience emerged as the only significant predictor. Pharmacists with 5–10 years of experience showed marginally significant (*p* = 0.054) fewer errors than the most experienced group (>20 years). Pharmacists with 10–15 years of experience had a statistically significantly lower number of errors compared with those with more than 20 years of experience (*p* = 0.035). No statistically significant differences were observed for pharmacists with 5–10 years of experience (*p* = 0.054) or 15–20 years of experience (*p* = 0.053) compared with the >20 years group.

### 2.3. Pharmacists’ General Knowledge and Attitude Towards Antimicrobial Resistance

Almost all participants answered that they know what antibiotic resistance means. It is noteworthy that 99% (*n* = 205) stated that they know what antimicrobial resistance is, while only 1% (*n* = 2) reported being unaware of it. Even more, 86% (*n* = 178) believe that the phenomenon of antimicrobial resistance is significant in Greece. In contrast, 8.7% (*n* = 18) stated that it is not a serious problem, and 5.3% (*n* = 11) did not respond or had no opinion.

**Figure 2:**
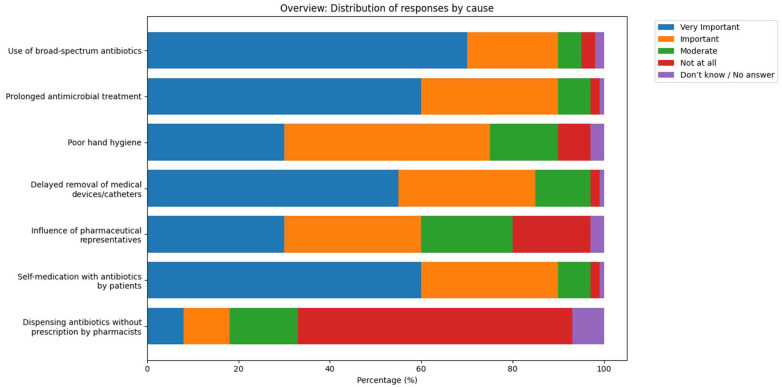
Pharmacists’ perceptions of the main causes of antimicrobial resistance (AMR). Respondents rated each factor on a five-point Likert scale ranging from “Not at all” to “Very important.” The most frequently cited factors included the use of broad-spectrum antibiotics, prolonged duration of antimicrobial therapy, and patient self-medication.

**Figure 3:**
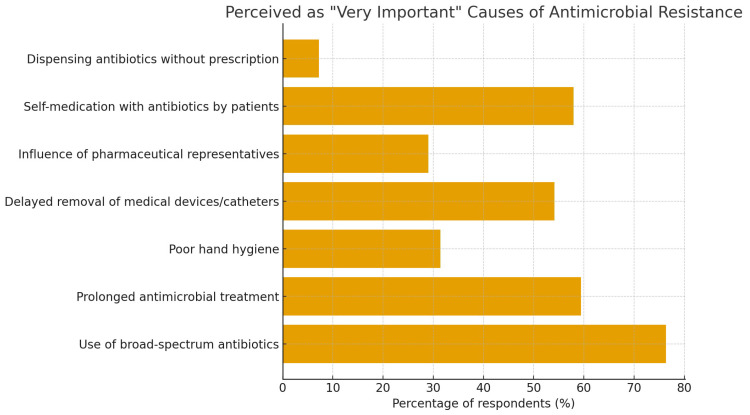
Percentage of respondents rating selected causes of antimicrobial resistance (AMR) as “Very important.” The use of broad-spectrum antibiotics was most frequently identified, followed by prolonged antimicrobial treatment, delayed removal of medical devices/catheters, and patient self-medication. The figure reflects pharmacists’ recognition of major clinical and behavioral drivers of antimicrobial resistance.

As shown in Figure 2, the most frequently cited contributing factor to increased antimicrobial resistance was the use of broad-spectrum antibiotics. Figure 3 shows the percentage of respondents who rated each cause of antimicrobial resistance as “Very Important”.

**Figure 4:**
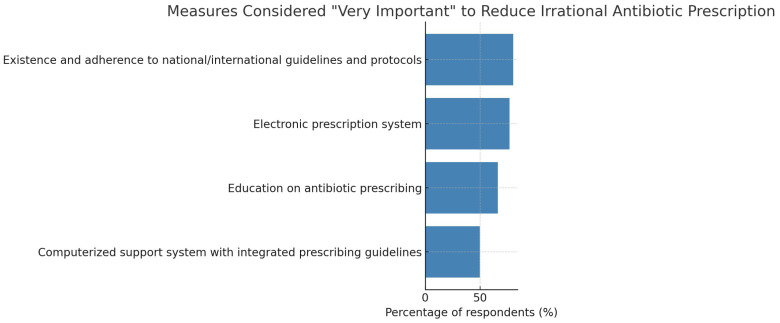
Respondents’ ratings of selected measures to reduce irrational antibiotic prescription as “Very important.” Bars represent the percentage of community pharmacists who rated each intervention as “Very important” on a five-point Likert scale. The most strongly endorsed measures were the existence and adherence to national/international guidelines and protocols and the use of electronic prescription systems, followed by education on antibiotic prescribing and computerized clinical decision-support systems. The figure highlights pharmacists’ support for guideline-based practice, digital prescribing tools, and continuous professional education as key strategies for promoting rational antibiotic use and combating antimicrobial resistance.

**Figure 5:**
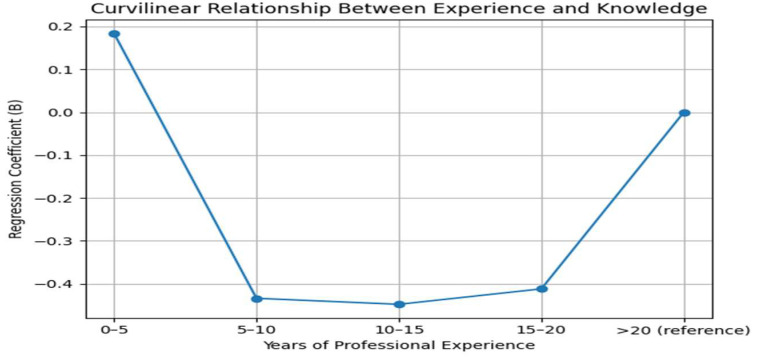
Curvilinear relationship between years of professional experience and knowledge regarding antibiotic use and antimicrobial resistance.

As presented in Figure 4, the most important cited measure for minimizing antimicrobial prescription was existence and adherence to national/international guidelines and protocols. All measures that were suggested by participants for controlling antimicrobial prescription and antimicrobial resistance are shown in Table 3 and Figure 5.

A multivariate logistic regression analysis was performed to identify factors associated with the knowledge regarding antimicrobial resistance. Male pharmacists provided more false responses than female pharmacists. However, this difference was only marginally significant (*p* = 0.059). Pharmacists with only a basic degree did not have a significantly different number of errors compared to those with a postgraduate degree (*p* = 0.847). Educational level is not a predictor of AMR knowledge in this sample. Pharmacists with 10–15 years of experience had a statistically significant and substantially lower number of errors than those with >20 years of experience (*p* = 0.035). Similarly, the 15–20 years’ experience group showed significantly fewer errors than the >20 years group. The model suggests a curvilinear relationship between experience and knowledge. Knowledge appears to improve and peak at the mid-career stage (10–20 years of experience) and then declines among the most senior pharmacists (those with over 20 years of experience), who show the highest rate of misconceptions (Table 4).

## 3. Discussion

Antibiotic resistance and stewardship are recognized as key indicators of healthcare system quality. This study examined the knowledge and attitudes of pharmacists in Achaia, Western Greece, regarding antibiotic use and antimicrobial resistance (AMR). The findings reveal a high level of awareness among pharmacists about both the appropriate use of antibiotics and the public health threat posed by AMR. Most notably, our findings demonstrate a curvilinear relationship between professional experience and knowledge, with mid-career pharmacists (10–20 years of practice) exhibiting significantly fewer misconceptions than both early-career and very senior pharmacists. This pattern, which has rarely been reported in the international literature, suggests that knowledge acquisition and retention regarding antimicrobial resistance may peak during mid-career and decline later if not reinforced through structured continuing professional development. This observation highlights the need for differentiated educational strategies that not only support newly qualified pharmacists but also actively update and re-engage senior professionals, whose foundational training may predate modern antimicrobial stewardship principles.

In accordance with international findings [4,5,16], most pharmacists correctly identified that antibiotics are not indicated for common viral infections such as sore throat, bronchitis, influenza, and diarrhea. However, knowledge gaps persist in relation to upper respiratory tract infections like rhinitis and sinusitis, where a notable proportion of respondents still considered antibiotics appropriate. Similar misconceptions have been documented in previous studies from Jordan, Eritrea, and the United Kingdom [4,5], underscoring the need for continued education to help pharmacists distinguish between bacterial and viral infections in routine practice. Even more similar to our study, Zajmi et al. (Kosovo) found that 42.5% believed antibiotics treat viral infections [17]. Moreover, Lagunen et al. reported that Greek citizens were more educated and knowledgeable about antibiotics (58.5% of Greeks and 44.2% of Turks identified antibiotics correctly), their effects (20.9% of Greeks and 26.3% of Turks agreed with wrong statements about antibiotics) and the risks of antibiotic resistance, compared to those from Turkey [18].

While previous studies in Europe have demonstrated that pharmacists possess good theoretical knowledge of AMR, daily practice is often shaped by patient expectations and insufficient regulatory [18]. Our results support that the dispensing of antibiotics without a prescription was cited as a major contributor to AMR, despite legal restrictions. These findings align with earlier research indicating that this practice remains common in Greece [19]. Moreover, our findings align with other studies in the broader Eastern Mediterranean region indicating that community pharmacists possess reasonable awareness of antimicrobial resistance but face challenges in optimal stewardship practices. For instance, AlAhmad et al. reported varied antimicrobial stewardship activities among community pharmacists in the United Arab Emirates, with gaps in the implementation of stewardship principles despite general awareness of AMR. These regional comparisons underscore the need for strengthened education and regulatory support across similar settings [20].

Multivariate logistic regression analysis revealed that pharmacists with 5–10 years of professional experience were significantly less likely to believe that antibiotics are always effective, compared with those with less than two years of experience. This suggests that clinical exposure and accumulated professional judgment contribute to improved understanding of antibiotic efficacy. Similar associations between professional maturity and better antibiotic-related knowledge have been reported in prior studies too [4,5]. Moreover, Isah et al. (Nigeria) also noted that factors such as education level, urban residence, and age influenced knowledge and attitudes toward antibiotics [21].

Nearly all participants (99%) reported awareness of antimicrobial resistance, and a majority (86%) perceived it as a major public health concern in Greece. These findings are consistent with international surveys of healthcare professionals [4,5,14,22]. However, as noted in other studies, awareness does not necessarily translate into appropriate practice. Despite their knowledge, pharmacists may still face contextual pressures to dispense antibiotics inappropriately, particularly in settings where antibiotics are readily available without prescription—a persistent issue in Greece and other Southern European countries. Qualitative evidence from neighboring countries also highlights systemic and behavioral factors influencing rational antibiotic use. In a multinational qualitative study, Özcebe et al. (2022) reported that both physicians and pharmacists in Turkey emphasized patient expectations, time pressure, and regulatory context as key challenges to rational antibiotic use—findings that are consistent with the contextual pressures reported by pharmacists in our study [23].

Furthermore, the findings of our study showed that the most seasoned pharmacists, those with over two decades of experience, hold significantly more misconceptions about antimicrobial resistance than their mid-career colleagues. This suggests that knowledge acquired during formal education and early career may become outdated if not reinforced by continuous professional development. It is possible that the foundational training of more senior pharmacists occurred before AMR became a prominent global health priority and before modern treatment guidelines were fully established. Targeted continuing education and accreditation programs are strongly recommended for senior pharmacists to update their knowledge of antimicrobial use and resistance patterns. Estevez et al. reported that the CPD framework may be a useful approach to support pharmacist development in hospital and health system settings and facilitate performance reviews [24]. These programs should be designed to address specific, persistent misconceptions. The lack of a significant effect from postgraduate education suggests that current specialized curricula may not sufficiently emphasize AMR, or that knowledge decays over time without practice. Integrating robust, mandatory AMR modules into both undergraduate and postgraduate pharmacy curricula, as well as into lifelong learning requirements, is crucial.

Although knowledge levels among pharmacists were generally adequate, the persistence of non-prescription antibiotic supply indicates a concerning gap between knowledge and practice. This discrepancy suggests that awareness alone is insufficient to ensure rational antibiotic dispensing. Several contextual factors may explain this inconsistency, including strong patient expectations and weak regulatory enforcement that normalize antibiotic access without prescription. Pharmacists, even when aware of the risks associated with inappropriate antibiotic use, may feel pressured to satisfy patient demands or maintain customer loyalty, particularly in competitive community settings. Moreover, limited inspection mechanisms and the lack of consistent penalties for non-compliance diminish the perceived importance of adhering to prescription-only regulations. Similar findings have been reported in previous studies, which identified the gap between theoretical knowledge and daily professional behavior among pharmacists, influenced by social, cultural, and economic pressures [14,18,24,25]. This finding underscores the importance of implementing systemic policy measures—such as stricter monitoring, public awareness campaigns, and the integration of pharmacists into antimicrobial stewardship programs that empower them to balance patient expectations with evidence-based practice [25,26].

Participants identified broad-spectrum antibiotic use, prolonged antimicrobial therapy, and patient self-medication as the primary drivers of resistance. These views reflect current scientific consensus [1,3,5] and align with priorities identified by international health authorities. For instance, a face-to-face cross-sectional survey by Zajmi et al. [17] reported that 25% of participants had used antibiotics without a prescription. The most common indications were flu (23.8%), sore throat (20.2%), and common cold (7.6%). Alarmingly, 42.5% of respondents in that study believed antibiotics were effective against viral infections.

In terms of proposed strategies to reduce AMR, participants emphasized the importance of antibiotic stewardship programs, adherence to prescribing guidelines, and increased education for healthcare professionals and the public. These findings highlight the essential role of pharmacists in national efforts to curb antibiotic resistance. Moreover, Greece has developed a National Action Plan on Antimicrobial Resistance (AMR) within the *One Health* framework, entitled “*One Health National Action Plan for Antimicrobial Resistance 2019–2023*” [Hellenic Ministry of Health, 2019; WHO, 2023]. Its main objectives include the strengthening of AMR surveillance systems across healthcare and veterinary sectors, promotion of rational antibiotic use, enhancement of prescription control mechanisms, and implementation of educational and awareness initiatives targeting healthcare professionals (including pharmacists) and the public.

Nevertheless, several systemic barriers may limit pharmacists’ full engagement in stewardship efforts. These include limited access to standardized prescribing protocols, insufficient collaboration between pharmacists and physicians, and weak enforcement of prescription-only regulations. Pharmacists are well-positioned to lead by example in AMR-reduction initiatives [19]. Closer collaboration with physicians, nurses, and infection control teams could ensure antibiotic use is aligned with clinical guidelines. Participation in interdisciplinary rounds, patient education programs, and stewardship committees can further amplify pharmacists’ contributions.

Additionally, the integration of technology into pharmacy practice offers new opportunities. Tools such as electronic health records and clinical decision support systems can help pharmacists monitor prescribing trends and identify areas for intervention. Strengthening pharmacists’ roles through targeted training, deeper integration into primary care teams, and engagement in public awareness campaigns could meaningfully reduce inappropriate antibiotic use and slow resistance development [19].

This study has several important limitations. First, the assessment of dispensing practices was based exclusively on self-reported data rather than direct observation, prescription audits, or simulated patient methodologies. It is important to emphasize that all practice-related data were self-reported. As such, the identified discrepancies between knowledge and reported behavior may not accurately reflect real-world dispensing practices. Self-report measures are subject to recall and social desirability bias and may lead to underestimation of inappropriate practices. Therefore, any policy implications drawn from these findings should be interpreted with caution. Consequently, the findings related to inappropriate antibiotic dispensing reflect pharmacists’ reported behaviors and attitudes and may be subject to recall bias and social desirability bias, potentially leading to underestimation of inappropriate practices. Second, the cross-sectional design precludes causal inferences. Third, the study was conducted in a single geographic region, which may limit generalizability. Moreover, as such, the findings reflect local community pharmacy practice and should not be generalized to Greece as a whole. Regional differences in healthcare access, enforcement of prescription regulations, and socio-economic conditions may lead to substantially different patterns elsewhere. Future research incorporating objective measures of dispensing behavior, such as mystery shopper studies or prescription data analysis, would provide more robust evidence regarding actual community pharmacy practices.

In conclusion, community pharmacists in Western Greece demonstrated high awareness of antibiotic use and antimicrobial resistance; nevertheless, clinically relevant knowledge gaps persist, particularly regarding antibiotic indications for predominantly viral infections. These findings identify specific areas where community pharmacy practice may be further supported. Targeted, experience-sensitive educational strategies and the structured involvement of pharmacists in antimicrobial stewardship initiatives may help strengthen rational antibiotic use in the community. Given that practice-related findings were self-reported, future research incorporating objective measures of dispensing behavior is essential to more robustly inform antimicrobial stewardship policies and interventions.

## 4. Materials and Methods

### 4.1. Study Design and Participants

This was a cross-sectional observational study conducted among community pharmacists in the region of Achaia, Western Greece, where approximately 25,000 residents are registered. A structured, self-administered questionnaire was distributed to all 350 pharmacists who are registered with the Pharmaceutical Association of Achaia and were randomly invited via emails and frequent reminders to participate voluntarily over a three-month period (May–July 2023). The potential participants were registered with the local Pharmaceutical Association of Achaia. All participants were provided with a brief overview of the study’s purpose and objectives prior to enrollment.

### 4.2. Questionnaire Development

The questionnaire was developed by independent members of the research team, informed by recent and relevant literature on antibiotic use and antimicrobial resistance [3,4,14,15]. Variables were selected a priori based on biological plausibility and existing literature. To ensure content validity, clarity, and internal consistency, a pilot study was conducted with a sample of 20 pharmacists. Their feedback was used to revise and finalize the instrument.

Internal consistency reliability was assessed using Cronbach’s alpha coefficient. The knowledge section of the questionnaire demonstrated acceptable internal consistency (Cronbach’s α = 0.73). The remaining sections (self-reported practices and antimicrobial resistance knowledge and attitudes) consisted mainly of dichotomous and conceptually heterogeneous items and were therefore treated as independent descriptive variables rather than psychometric scales; consequently, internal consistency indices were not applicable to these sections.

The final version of the questionnaire, written in Greek, consisted of four sections: Part A: Demographic information (e.g., age, gender, years of professional experience, and employment sector), Part B: Knowledge of antibiotic use, including understanding of antibiotic effectiveness in common infections (e.g., sore throat, bronchitis, rhinitis), Part C: Self-reported practices related to antibiotic administration (eight questions), Part D: Knowledge and attitudes toward antimicrobial resistance (seven questions).

### 4.3. Ethical Considerations

The study received approval from the University Ethics Committee (Approval Number: 855/27 March 2023) and was conducted in accordance with the principles of the Declaration of Helsinki. Reporting followed the STROBE (Strengthening the Reporting of Observational Studies in Epidemiology) guidelines.

### 4.4. Statistical Analysis

Data analysis was performed using IBM SPSS Statistics, version 27 (IBM Corp., Armonk, NY, USA). A significance level of *p* < 0.05 was adopted for all inferential analyses.

Descriptive statistics were used to summarize demographic variables and responses related to knowledge, attitudes, and practices regarding antibiotic use and antimicrobial resistance. Associations between demographic variables and knowledge outcomes were assessed using the Chi-square test of independence. Post hoc analyses were conducted to explore statistically significant differences across study groups. Additionally, multivariate logistic regression analysis was used to identify predictors of misconceptions, such as the belief that antibiotics are always effective. For the regression analyses, categorical variables were dummy-coded. Reference categories were selected a priori based on conceptual relevance and interpretability. Pharmacists with more than 20 years of professional experience were used as the reference group, as they represent the most experienced cohort and provide a meaningful benchmark for comparisons across earlier career stages. This approach enabled the assessment of whether the likelihood of misconceptions differed among early- and mid-career pharmacists relative to the most professionally experienced group. In the multivariate logistic regression analysis, several demographic and professional factors were included. Specifically, the model adjusted for age, gender, years of professional experience, and educational level, to account for potential confounding effects. These variables were selected based on their potential influence on knowledge and attitudes toward antibiotic use.

## Figures and Tables

**Table 1:** Demographic characteristics of participants.

Variable	Categories	Frequency (N)	Percentage (%)
Gender	Male	98	47.3
	Female	109	52.7
Age group	18–30 years	37	17.9
	31–40 years	57	27.5
	41–50 years	81	39.1
	51–65 years	29	14.0
	>65 years	3	1.4
Educational level	Secondary education	0	0
	Tertiary education	118	55.6
	Master’s degree	76	36.7
	Doctorate (PhD)	13	7.7
Work sector	Private	207	100.0
	Public	0	0
Years of professional experience	0–2 years	19	9.2
	2–5 years	23	11.1
	5–10 years	40	19.3
	10–15 years	45	21.7
	15–20 years	56	27.1
	>20 years	24	11.6

**Table 2:** Associations between demographic factors (gender, age, educational level, and years of professional experience) and the level of knowledge regarding antibiotic use.

Parameter	B	Std. Error	95% Wald Confidence Interval (Lower)	95% Wald Confidence Interval (Upper)	Wald Chi-Square	df	Sig.
(Intercept)	0.466	0.2000	0.074	0.857	5.422	1	0.020
Male/Female	0.252	0.1334	−0.009	0.514	3.577	1	0.059
Basic education	−0.024	0.1256	−0.270	0.222	0.037	1	0.847
Work experience 0–5 years	0.184	0.1957	−0.200	0.567	0.881	1	0.348
Work experience 5–10 years	−0.434	0.2250	−0.875	0.007	3.725	1	0.054
Work experience 10–15 years	−0.448	0.2125	−0.864	−0.031	4.438	1	0.035
Work experience 15–20 years	−0.412	0.2128	−0.829	0.005	3.750	1	0.053

**Table 3:** Distribution of respondents’ ratings on measures to reduce irrational antibiotic prescription. Respondents rated each factor on a five-point Likert scale ranging from “Not at all” to “Very important.” The most important cited measures included the existence and adherence to national/international guidelines and protocols and the use of electronic prescription systems.

Measure	Very Important	Important	Moderate	Not at All	Do Not Know/No Answer
Education on antibiotic prescribing	66.18% (*n* = 137)	18.84% (*n* = 39)	12.56% (*n* = 26)	2.42% (*n* = 5)	—
Existence and adherence to national/international guidelines and protocols	80.19% (*n* = 166)	15.94% (*n* = 33)	3.38% (*n* = 7)	0.48% (*n* = 1)	—
Electronic prescription system	76.81% (*n* = 159)	19.32% (*n* = 40)	2.42% (*n* = 5)	1.45% (*n* = 3)	—
Computerized support system with integrated prescribing guidelines	49.76% (*n* = 103)	18.36% (*n* = 38)	19.81% (*n* = 41)	10.14% (*n* = 21)	0.97% (*n* = 2)

**Table 4:** Parameters associated with the knowledge regarding antimicrobial resistance.

Parameter	B	Std. Error	Lower 95% CI	Upper 95% CI	Wald Chi-Square	Sig.
Male	0.252	0.1334	−0.009	0.514	3.577	0.059
Female	0a	—	—	—	—	—
Basic Education	−0.024	0.1256	−0.270	0.222	0.037	0.847
Postgraduate Studies	0a	—	—	—	—	—
Work Experience 0–5 years	0.184	0.1957	−0.200	0.567	0.881	0.348
Work Experience 5–10 years	−0.434	0.2250	−0.875	0.007	3.725	0.054
Work Experience 10–15 years	−0.448	0.2125	−0.864	−0.031	4.438	0.035
Work Experience 15–20 years	−0.412	0.2128	−0.829	0.005	3.750	0.053
Work Experience > 20 years	0a	—	—	—	—	—

## Data Availability

The original contributions presented in this study are included in the article. Further inquiries can be directed to the corresponding author.
